# Regulatory aspects of biological medicines in Bosnia and Herzegovina

**DOI:** 10.17305/bjbms.2021.6910

**Published:** 2022-03-23

**Authors:** Biljana Tubić, Saša Jungić

**Affiliations:** 1 Department of Pharmacy, Faculty of Medicine, University of Banja Luka, Banja Luka, Bosnia and Herzegovina; 2 Agency for Medicines and Medical Devices of Bosnia and Herzegovina, Banja Luka, Bosnia and Herzegovina; 3 Department of Medical Oncology, Faculty of Medicine, University of Banja Luka, Banja Luka, Bosnia and Herzegovina; 4 Clinic of Medical Oncology, University Clinical Centre Republic of Srpska, Banja Luka, Bosnia and Herzegovina

**Keywords:** Original biological medicines (originator biologics), biosimilars, regulatory approach, interchangeability, safety

## Abstract

The use of the biological medicines, also called “biologics,” has contributed to the progress of the treatment of many chronic diseases, such as cancer, rheumatoid arthritis, Crohn’s disease, multiple sclerosis, and psoriasis. However, biologicals are expensive for healthcare systems in several countries. Their availability has been a global issue, which has affected many patients that suffer from various diseases. A biosimilar medicine, also called “biosimilar,” is a medicine with similar characteristics in terms of quality, biological activity, safety, and efficacy as the approved original biological medicine, known as “originator biologic.” Biosimilars generate competition within the market because they lower the prices of biologics and thus allow for an increase in patient access. However, there are barriers when it comes to the acceptability rate of biosimilars and how interchangeable they are with the originator biologic. In this review, we present a national regulatory framework for biologics along with its limitations, a system of monitoring the safety profile of biologics, the guideline for interchangeability, and a list of approved and available biologics in Bosnia and Herzegovina. Additionally, recommendations were made here in order to provide opportunities for greater acceptance of biosimilars and better access to biologics. These recommendations include, but are not limited to, strengthening the national regulatory framework for biologics, capacity building, increasing awareness among healthcare providers for reporting adverse drug events and active pharmacovigilance, and better definitions of interchangeability. Finally, awareness among healthcare providers regarding biosimilars and biologics should be raised through continuous education and workshops, and by including this important topic in the graduate and postgraduate curriculum programs in the country.

## INTRODUCTION

A biological medicine, also known as a “biologic”, is a ­medicine that contains a biologically active substance (biological macromolecule) from a biological source, such as living cells or organisms (human, animal, or plant), and microorganisms such as bacteria or yeast [1]. According to the United States Food and Drug Administration (FDA), biologics include a wide range of products such as vaccines, blood and blood components, allergens, somatic cells, gene therapy, tissues, and recombinant therapeutic proteins. Biologics can be composed of sugars, proteins, nucleic acids, or a complex combination of these substances. Biologics can even be living entities such as cells and tissues [2]. They offer treatment options for patients with chronic diseases such as diabetes, autoimmune diseases, cancers, rheumatoid arthritis, Crohn’s disease, multiple sclerosis, and psoriasis [1,3-5]. The use of biologics has contributed to the progress in the treatment of these diseases, and this area is of great importance for public healthcare at the global level.

Biologics are much more structurally complex than the small chemical molecules. Therefore, manufacturing process of biologics is more complex. They are often produced by biotechnological methods and other cutting-edge technologies using sophisticated cell systems and recombinant DNA technology, polymerase chain reaction (PCR), hybridoma technology, and lab-on-a-chip [1-3]. These technologies are one of the reasons biologics are more expensive compared to small chemical drugs. Biologics are frequently costly for healthcare systems globally and access to them may be a problem for many patients worldwide [6-13].

The basic principle of the pharmaceutical legal framework is to ensure a high level of health care by providing medicines of high quality, safety, and efficacy with adequate access through the competitiveness of the pharmaceutical industry on the market.

**FIGURE 1:**
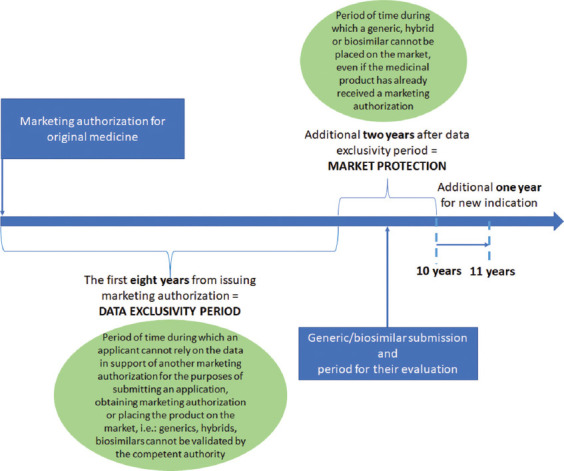
Data exclusivity period and market protection. “Without prejudice to the law on the protection of industrial and commercial property, medicinal products for human use which have been authorized in accordance with the provisions of this regulation shall benefit from an eight-year period of data protection and a ten-year period of marketing protection, in which connection the latter period shall be extended to a maximum of eleven years if, during the first eight years of those ten years, the marketing authorization holder obtains an authorization for one or more new therapeutic indications which, during the scientific evaluation prior to their authorization, are held to bring a significant clinical benefit in comparison with existing therapies” [15].

A period of eight years from the initial marketing authorization (MA) of an originator medicine, also known as an “originator”, is known as the data exclusivity period. During this period, the marketing-authorization holder (MAH) benefits from the exclusive rights to the results of preclinical tests and clinical trials on the originator medicine. After this period, the MAH is obliged to share this information with companies wishing to develop copy versions of the originator [14]. The data exclusivity period is followed by two more years, also called “market protection”. At the end of a ten-year period of marketing protection, generic medicines, also known as “generics”, and biosimilar medicines, also known as “biosimilars”, are able to come to the market, as this period marks the end of the MAH benefits (Figure 1). A ten-year period can be extended to a maximum of eleven years if during the first eight years of those ten years, the MAH has obtained an authorization for one or more new therapeutic indications [15].

Generics and biosimilars are often offered at lower prices than the originator drug, potentially reducing costs for patients and the healthcare system [16]. The prices of originators decrease after the MAH loses market exclusivities [9]. Thus, reduction of costs could result in savings for the healthcare systems and increasing number of patients having the access to the necessary medicines [5,9,10,13,17-19]. Prices per treatment day (total market) have been reduced in almost all markets but to different degrees from −65% to 2%, due to a combination of factors. These factors include the level of competition, the acceptance of non-accessible market products (primarily differentiated by fewer injections), as well as development costs of originators and biosimilars [5].

**FIGURE 2:**
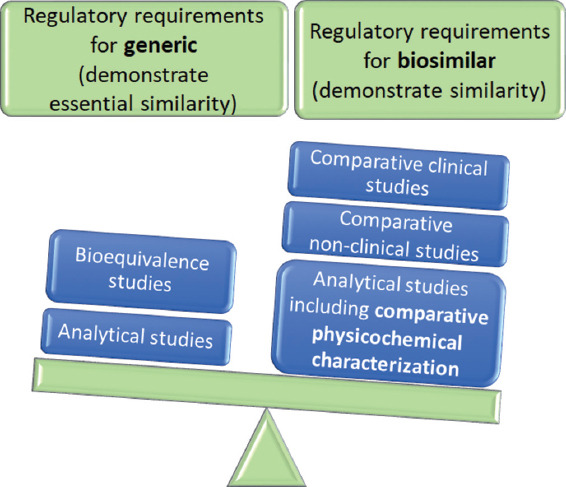
Comparison of regulatory requirements between generics and biosimilars.

Biologics are extremely sensitive to manufacturing conditions and therefore more difficult to characterize and produce than small chemical medicines. They are made by living organisms, which are naturally variable and the active substance in the final biologics can have an inherent degree of minor variability (microheterogeneity). A minor change in manufacturing may lead to differences in the structure, stability, or other quality aspects of the final product. All of these factors have the potential to affect tolerability and/or efficacy and increase the risk of immune responses through the appearance of anti-drug antibodies and allergic reactions. Therefore, this minor variability must fall within the acceptable range to ensure consistent quality, safety, and efficacy. This is done by adjusting the manufacturing process to guarantee that the active substance fits into the desired specifications range. This degree of minor variability can be present within or between batches of the same biological medicine [1,20]. Due to the fact that “the process is the product”, the regulatory approach is different for biosimilars than for generics [21], i.e., biosimilars meet the more demanding regulatory requirements than generics (Figure 2).

Generics are defined as essential similar small molecule drugs with the same qualitative and quantitative composition of the active substance, and the same pharmaceutical form as an already approved originator [22-24]. The essential similarity between an originator and a generic has been demonstrated by the appropriate bioequivalence studies.

Biosimilars are defined as similar biological medicines with a proven similarity in terms of quality, biological activity, safety, and efficacy as the already approved originator biologics [1]. Similarity between originator biologic and biosimilars is evaluated based on a “head-to-head” and “step-by-step” approach. It must be based on the extensive product characterization and an abridged non-clinical and clinical data package [1]. Regulatory bodies for medicines, such as the European Medicines Agency (EMA), FDA, the World Health Organization (WHO), and The International Council for Harmonisation of Technical Requirements for Pharmaceuticals for Human Use (ICH) have prepared a regulatory framework with many guidelines, which include the regulatory requirements for similarity assessment between originator biologics and proposed biosimilars in an approval process. The expertise of the regulatory competent authorities responsible for the licensing of biologics is of crucial importance for the appropriate evaluation [6].

The robustness of this regulatory framework ensures the maintenance of high levels of quality, safety, and efficacy of approved medicines while supporting innovation.

The success in treating various chronic diseases is highly dependent on the access to biologics, whereby biosimilars, with their lower market prices, provide the chance for increased accessibility. However, this is globally still not the case, and there are few major reasons why the attempt to broaden increased access has not been fully successful. One factor is the lack of competition in the market and a lack of sufficient uptake of biosimilars to generate price competition with their originator. There are several reasons for the variable uptake and utilization. These include the lack of incentives for healthcare professional to use biosimilars; lack of trust in biosimilars among healthcare providers and patients; similarity, but not essential similarity between originator biologics and biosimilars with the perception that biosimilars are not as good as the originator biologics; lack of clear guidance, government policies or clinical guidance with defined conditions for interchangeability of originator biologic with the biosimilars; possible appearance of unknown adverse effects; and the “innovator’s reach” by shifting towards patented alternatives and interfering with intellectual property rights [11,14,25-28]. A lack of incentive policy implementation with regard to biosimilar uptake and utilization or the variation in incentive policies in Europe or across the globe have also potentially influenced the market dynamics [29].

The main role of regulatory authorities is to ensure that only high-quality, safe, and effective biosimilars are available in the market. Experience with the introduction of generics has shown that gaining the trust of all stakeholders, including policy-makers, regulatory competent authorities, and healthcare professionals is essential for increasing access [30].

In this review, the results of the benchmarks investigation for biologics in Bosnia and Herzegovina (B&H) have been presented. The national legal framework and regulatory requirements, data exclusivity period, labeling and monitoring of safety profile, interchangeability, and a list of approved and available biologics for the B&H market were discussed.

## DATA SOURCES

The Agency for Medicinal Products and Medical Devices of Bosnia and Herzegovina (ALMBIH) is the regulatory competent authority at the state level. For the research of the national legal framework for biologics, we used the legal documents from the internet presentation of the Official Gazettes of B&H [31] and legal documents from the ALMBIH website [32]. Information regarding data exclusivity, labeling and monitoring of safety profile, and interchangeability of biologics was taken from the ALMBIH website [33] and compared with the European Union (EU) requirements published on the EMA website [34]. A list of the approved biologics for the B&H market was created by inquiring into the database at the ALMBIH [35]. Medicines are listed by their Anatomical-Therapeutic-Chemical classification [36].

Bosnia and Herzegovina has two entities and the Brcko District. The Federation of Bosnia and Herzegovina (Federation of B&H) and the Republic of Srpska (RS) are entities with their own constitutions. Each entity is responsible for maintaining health care within its territory. The RS has one health insurance fund, the Health Insurance Fund of the Republic of Srpska (HIF RS) [37]. On the other hand, the Federation of B&H consists of ten cantons, with each canton having its own health insurance fund. At the entity level of the Federation of B&H, there is the Health Insurance and Reinsurance Institute of the Federation of B&H (HIRI F&B) [38]. A list of biologics that are reimbursed by abovementioned health care insurance funds were downloaded from their websites.

Preventative vaccines and plasma-derived products, as well as their recombinant analogs, were excluded from the scope of this review article because the focus is on biologics, which contribute to the progression and treatment of many chronic diseases, such as cancer, rheumatoid arthritis, Crohn’s disease, multiple sclerosis, and psoriasis.

## SECTIONS ON POLICY OPTIONS FOR BIOLOGICS IN B&H

### Legal framework and requirements for licensing

The ALMBIH was established in 2009 as the sole competent authority for medicine and medical devices for the B&H market. One of its main responsibilities is to authorize medicines based on a positive assessment of quality, efficacy, safety (Q/E/S), and risk/benefit ratio. The evaluation of Q/E/S is prepared by the internal experts and experts from the Committee for Medicines for Human Use which functions at the state level [39]. Sometimes, during the process of issuing MA for radiopharmaceuticals, a new originator biologic, or biosimilar which has not been previously approved for the EU market, the ALMBIH assigns an expert from the List of experts of B&H. This list includes experts from B&H and former Yugoslavian countries [40]. Based on Article 20 paragraph 6 of the Medicinal Products and Medical Devices Act of B&H (Official Gazette of B&H, number 58/08), the director of ALMBIH, with the prior consent provided by the Professional Board, will endorse the List of experts B&H.

The legal framework for licensing medication for the B&H market is available in the Medicinal Products and Medical Devices Act of B&H (Official Gazette of B&H, number 58/08) [39] and the Rulebook on procedure and manner for issuing MA approval (Official Gazette of B&H, number 75/11) [41]. A fast-track procedure of issuing MA is established by Article 32 and Article 33 of that Rulebook. This procedure refers to medicines that have previously been approved for the EU market by the centralized procedure, decentralized procedure, or mutual recognition procedure. In such case, ALMBIH accepts an assessment report by the Committee for Medicinal Products for Human Use or an assessment report from the referent member state within the decentralized procedure or mutual recognition procedure. In a situation where the requirements stated in Articles 32 and 33 have not been met, ALMBIH itself makes an assessment based on Q/E/S.

A separate legal document with regulatory requirements for approval of biologics for the B&H market does not exist. The national regulatory framework for licensing is the same for both conventional pharmaceuticals and biologics, except for the regulatory requirements for biosimilars that are more extensive than regulatory requirements for generics. These are present in Article 27 of the Rulebook on procedures for issuing MA approval [41].

During the process of issuing MA for biologics, dossiers are reviewed and evaluated according to the specific EMA guidelines for active biological substances (e.g., monoclonal antibody, low-molecular-weight heparin, etc.), as well as the ICH, and WHO guidelines. Assessment of quality, comparable non-clinical and clinical studies, is performed in accordance with the EMA requirements. These requirements include demonstrating the similarity between originator biologic and proposed biosimilar in terms of quality, safety, and efficacy. Immunogenicity studies are also required prior to approval. Only an originator biologic approved on the basis of a complete registration dossier (complete Q/S/E data) can serve as a comparator for head-to-head studies with the proposed biosimilar to demonstrate similarity in terms of Q/E/S [6,41]. Dosage form, strength, and route of administration of the biosimilar should be the same as for the originator biologic. A comprehensive assessment of the quality characterization of the originator biologic and biosimilar is a prerequisite for the reduction of non-clinical and clinical data required for authorization.

The licensing procedure of an originator biologic or biosimilar for the B&H market does not require previous assessment performed by the EMA or approval issued by the European Commission (EC) through the centralized procedure [24,39]. This is in contrast with the regulatory requirement in the EU member states [24], as well as the countries of the East European region (Bulgaria, Romania, Slovenia, Croatia, Serbia, Montenegro, and the Republic of North Macedonia). Since the licensing procedure in BIH does not resemble licensing procedures of neighboring countries, it becomes a considerable challenge for ALMBIH.

The ALMBIH has an insufficient number of highly educated healthcare employees (Master Pharmacy degree and Medical Doctor degree) – this has remained to be true since its establishment. This is a consequence of employment discontinuation at the state level and lack of independence of the ALMBIH (it is financed from the budget of B&H and is part of the state administration) [39]. Therefore, the staff and the lack of independence remain to be the reason for undeveloped internal expertise. Due to the lack of internal experts for the assessment of Q/E/S of biologics, the ALMBIH has to assign an expert from the list of experts B&H.

**FIGURE 3:**
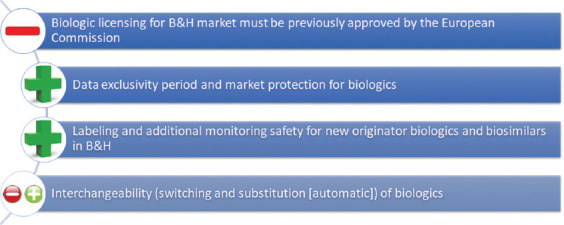
Compliance between the B&H and EU regulatory requirements for biologics −: Insufficient harmonization; +: Harmonization requirements; −+: No unique requirements at the level of EU; B&H: Bosnia and Herzegovina; EU: European Union.

A comparison between the B&H and EU regulatory requirements for biologics is given in Figure 3.

### Data exclusivity period

The data exclusivity period of eight years and the market protection period of two years have been defined as ten years in the EU (Figure 1). In the United States, the data exclusivity period for small molecule drugs has been defined as 7 years [42] and 12 years for biological medicines [6,43].

Based on the Article 34, paragraph 1, point (c) of the Medicinal Products and Medical Devices Act of B&H (Official Gazette of B&H, number 58/08), the data exclusivity period in B&H is defined as 8 years with additional 2 years for the market protection period, which is the same as the EU. The data exclusivity period and the market protection period are mandatory for issuing MA and apply to the B&H market generics and biosimilars only from July 2021. Previously, it was not mandatory to follow the data exclusivity and market protection period based on the Article 141 of the Medicinal Products and Medical Devices Act of B&H [39], and decisions of the Professional Board (Official Gazette of B&H, number 57/13 and number 75/18) [44,45].

### Labeling and monitoring of safety profile

Since January 2018, all biological medicines (originals and biosimilars) approved by ALMBIH ten or less years ago, as well as all newly approved biologics, have been put under additional monitoring on the B&H market. These medications are labeled with a black upside-down triangle (▼) on the packaging. Additionally, the medication comes with a Patient Information Leaflet and a Summary of Product Characteristics (SmPC) in one of tree official languages in B&H (Bosnian, Croatian or Serbian) [33]. This symbol is an alert to all healthcare professionals to closely monitor biologics, be more vigilant with these medicines, and to report any suspected adverse drug reactions (ADR) [1]. For each biosimilar approved for the B&H market, there is information in the SmPC that the biologic the patient is using is similar to an appropriate originator biologic.

Regarding the information presented above, monitoring the safety profile of biologics during the post-marketing period (especially of biosimilars) is emphasized more than with the other available medications on the B&H market. In 2019, ALMBIH became a full member of the Uppsala Monitoring Centre – WHO. Although no online reporting solution for healthcare professionals and MAHs has yet been developed to date, ALMBIH has created a guide for detecting and reporting the ADRs with certain specific requirements for monitoring the ADRs of biologics. Each report form should provide a brand name, International Nonproprietary Name (INN), batch number, and all the other facts critical for traceability of the product [33,46]. Reporting forms and educational materials are available on the ALMBIH website [47]. However, during the period 2011-2018 year, the number of the reported ADRs in B&H was lower compared to the other nearby countries such as Croatia, Serbia, Montenegro, and Slovenia [48]. In the Department of Pharmacovigilance, which is an organizational unit of the ALMBIH, there are only three employees (one Master Pharmacy degree and two technical employees). The lack of highly educated healthcare employees in the department is the reason for lack of causality assessments and it holds responsibility for the insufficient development of the pharmacovigilance system in B&H. Additionally, insufficient education in the field of pharmacovigilance and lack of knowledge of healthcare professionals on how the pharmacovigilance system in B&H functions (whom to report and where to find an ADR reporting form) are also reasons for underreporting in B&H [48,49]. Due to these facts, all the safety information on biologics and other medicines are taken from EMA.

### Interchangeability (switching and substitution)

Interchangeability of biologics is a responsibility of each individual country. The EMA’s definition of interchangeability is as follows: “Interchangeability of biologics refers to the possibility of exchanging one biologic for another one that is expected to have the same clinical effect. This could mean replacing an originator biologic with a biosimilar (or vice versa) or replacing one biosimilar with another. A replacement can be done by:

- Switching, which is when the prescriber decides to exchange one biologic for another biologic with the same therapeutic intent.
- Substitution (automatic), which means obtaining an interchangeable biologic at the pharmacy level without consulting the prescriber” [1].

The FDA has presented a different definition of interchangeability: “The biological product may be substituted for the reference product without the intervention of the health-care provider who prescribed the reference product, thus corresponding to the EMA’s definition of automatic substitution” [12].

The EMA does not make a distinction between biosimilars and interchangeable products. In fact, the EMA has abstained from taking an official position on the interchangeability of biosimilars. In the USA, a distinction is made between interchangeable products and biosimilars, which is defined in the Biologics Price Competition and Innovation Act of 2009 [29].

There is no government policy document for regulating switching and substitution of biologics in B&H to date. However, switching of biologics (between originator and biosimilar) is recommended by ALMBIH and requires appropriate monitoring [33]. The prescribing physician is responsible for this decision, while pharmacists dispense the prescribed biologics. Uncontrolled exchange between biologics without adequate clinical monitoring must be avoided. If this exchange takes place, detailed product and batch information must be recorded in the patient file to ensure the traceability of the product in the event of problems (appearance of ADR, e.g., an allergic reaction) [33,50]. Substitution of biologics in B&H has not been recommended yet.

Contrary to generics, in most European countries, automatic substitution of biosimilars is neither allowed nor recommended [13]. Based on the experience with the introduction of generics, it can be assumed that the clearly defined guideline for interchangeability (switching and substitution) would benefit the uptake of biosimilars, and in this way stimulate competition in the market.

### Approved biologics and availability of biologics

A list of the approved biologics (by INNs) for the B&H market [35] is presented in Table 1. Biologics approved by the ALMBIH without evaluation by the EMA and approval by the EC are marked with an asterisk in Table 1. This includes the originator biologic CIMAvax-EGF^®^ (conjugated rEGF-rP64K) which is produced by the Center of Molecular Immunology in Havana, Cuba (approval expired in 2021, and has not been renewed). In addition, there is a biosimilar Herticard^®^ (rituximab) and a biosimilar Acellbia^®^ (trastuzumab) which are both produced by the Russian biotechnology company BIOCAD. There is a biosimilar GENSULIN^®^ (human insulin), which is produced by Bioton that is located in Poland. The approval for Herticard^®^ was withdrawn in 2021 by the MAH.

**TABLE 1:**
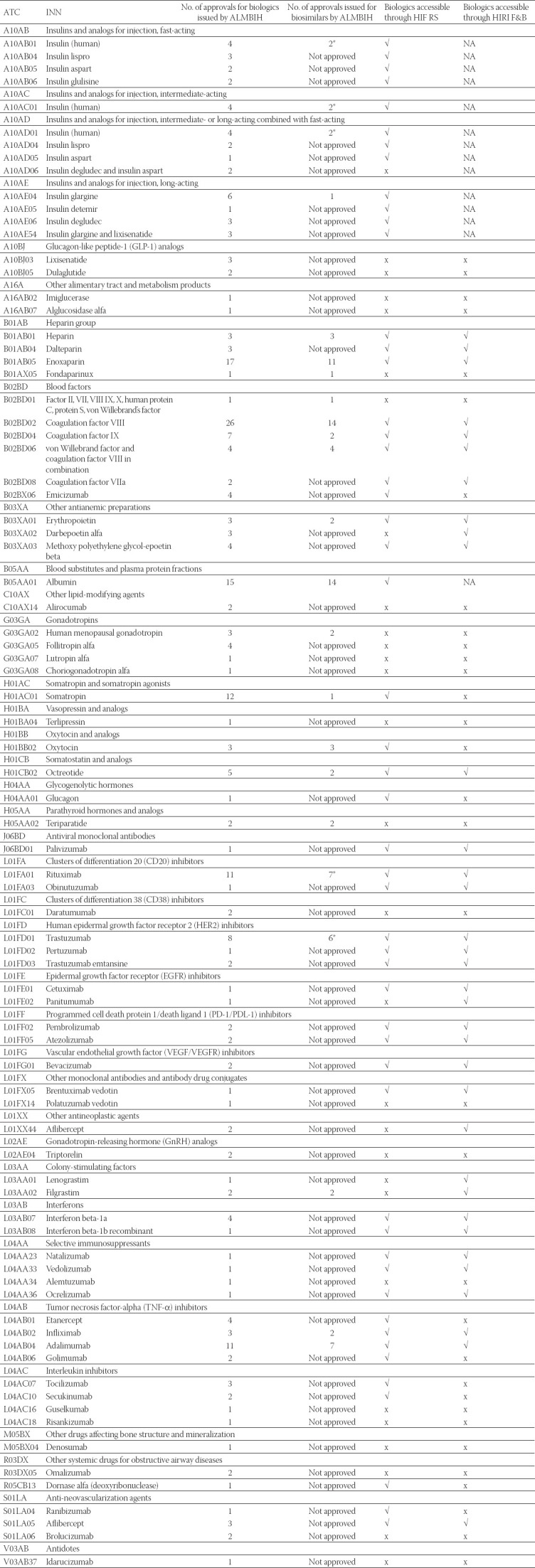
List of approved biologics comparing with biologics reimbursed by the most important B&H health-care funds (approved and available biologics)

Only biologics included in the list of medicines, which are reimbursed by health insurance funds, are accessible to patients. Lists of biologics which are reimbursed by the HIF RS and HIRI F&B are shown in Table 1. These medications are procured only centrally, at the level of the responsible health insurance fund, within tender procedures.

## ACTIONABLE RECOMMENDATIONS

### Clearly define and adopt a legal framework and the requirements for licensing of biologics

Biosimilars elicit a greater chance for broader accessibility of expensive biologics, for example, monoclonal antibodies, to patients within the health insurance system in B&H and worldwide. Expertise in regulatory assessment of biologics is essential for ensuring quality, safety, and efficacy during the licensing of the product and what happens to the product post-licensure and when it is distributed into the market. The role of the regulatory authority is very important, however, the regulatory framework and regulatory requirements for biologics in B&H are insufficient and not clearly defined. The ALMBIH has not developed internal expertise for the biologics. Due to the infrastructure currently in place, this has caused a lack of trust in the biologics approved by ALMBIH [51,52]. With respect to this, it is necessary to strengthen the regulatory system for biologics in B&H by adopting national guidelines and policy documents that are in line with EU regulations. The EU guidelines provide and present requirements for individual classes of biologics. Also, best experts in this field are included in the process of assessing and issuing MAs for biologics.

### Develop an internal expertise for biologics within the ALMBIH

ALMBIH should aim to employ highly educated professionals to become the internal expertise for biologics – increase capacity, experience, and competency in the assessment of Q/S/E and risk/benefit ratio for biologics. Gaining the trust in the assessment of biosimilars performed by ALMBIH is one of the main challenges that is in the way of accepting the biosimilars as an option for increasing biologics access in the healthcare system. The decision to approve the biosimilars should go beyond mere cost-savings opportunity, as quality and patient safety must also be important. When biosimilars are approved, they need to be comparable to the appropriate originator biologics. They should meet the determined standards for quality, efficacy, and patient’s safety. Therefore, assessment of biologics in the process of issuing MA by ALMBIH must be appropriate and in accordance with all EU, ICH, and WHO standards.

### Improve the pharmacovigilance monitoring system

Biologics on the B&H market are under additional monitoring and they are labeled with the ▼ sign. However, the pharmacovigilance program in B&H is insufficient to create an accurate database containing data on each biologic’ clinical use, including patient registries and retrospective or prospective observational post-marketing studies. ALMBIH should improve their pharmacovigilance system in collaboration with other stakeholders. Online ADR reporting is one method that can increase the reporting rate. This method was primarily proposed by pharmacists. ALMBIH should popularize ADR reporting by providing additional education, training, and information to the healthcare professionals about this issue [48]. It should employ new highly educated professionals with the necessary experience and competency to perform assessments regarding the safety profile of biologics. The lack of highly educated employees (pharmacists and medical doctors) could be temporarily resolved by the engagement of a committee with experts from B&H who can introduce and establish a causality assessment.

### Establish national conditions for interchangeability (switching and substitution)

The interchangeability (switching and substitution) of biologics is not clearly defined in B&H. Health-care professionals and other stakeholders in B&H need a government policy document that would precisely determine the conditions for switching and performing substitutions of originator biologics with the biosimilars and between biosimilars. By strengthening the Agency’s internal expertise for biologics and improving pharmacovigilance system, the automatic substitution of originator biologic with biosimilars should be recommended.

### Improve the accessibility of biosimilars (promote biosimilar uptake)

Except for advanced stem cell and gene therapy which are not approved in the B&H market, the range of the licensed biologics for the B&H market is otherwise satisfactory (Table 1). However, the previous studies reported the limited accessibility of biologics and the limited use of biosimilars in B&H [9,10,52]. An important way to improve the accessibility of biologics in B&H is to put the important biologics on the list of reimbursed medicines. Education directed at healthcare professionals and key physicians would help fill the knowledge gaps, improve confidence in biosimilars, and increase biosimilar prescribing [53]. In order to raise awareness and knowledge regarding biologics among healthcare professionals, this topic should be included in medical and pharmacy graduate and post-graduate curricula by introducing subjects such as pharmaceutical biotechnology, clinical use of biologics, the role of the pharmacist, regulation of biologics, etc. Several large surveys have shown a lack of knowledge and trust in biosimilars among patients and other stakeholders (policy-makers and payers) [25,26]. It is important to set up a close collaboration among all stakeholders to communicate, develop, and disseminate factual information about biosimilars. The European Society for Medical Oncology has developed a nuanced approach emphasizing the role of Health Technology Assessment processes as well as the need to address the economic impact of high-cost medications on long-term sustainability [54]. According to Knežević and Griffiths [21], robust manufacturing processes with many in-process quality controls and consistency of quality from a lot-to-lot are important features for the efficacy and safety of the biologics. As previously emphasized, consistency is crucial because minor changes can lead to major adverse effects of biologics, such as immunogenicity, with potentially serious clinical implications [27]. Consequently, one of the new specific regulatory requirements for approving biologics should be a high level of consistency in the manufacturing process with more frequent in-process controls.

## CONCLUSION

The lack of the policy document which would precisely determine the conditions for licensing and interchangeability of biologics, lack of internal expertise within ALMBIH, and underdeveloped pharmacovigilance system in B&H contribute to the lack of wider trust in biosimilars by healthcare professionals, patients, and stakeholders. This situation and consequently better accessibility of biologics in B&H could be improved by:

- Adopting the clearly defined regulatory framework and requirements, as well as related terminology for originator biologics and biosimilars,
- Increasing internal capacity, expertise, and competency in the assessment of Q/E/S and risk/benefit ratio of biologics,
- Encouraging a process of ADRs reporting through permanent education and training,
- Establishing the causality assessment for ADR reports,
- Adopting the government policy document with well-defined conditions for interchangeability of biologics,
- Raising the awareness of healthcare professionals about biosimilars, and biologics in general including this topic in the graduate and postgraduate curriculum programs.

Finally, a collaboration with other national regulatory authorities and the WHO should be ensured in order to promote and implement a global approach to the regulation of biologics.

## References

[1] European Medicines Agency. Biosimilar Medicines:Overview.

[2] U.S. Food and Drug Administration What Are “Biologics”Questions and Answers.

[3] European Medicines Agency New Guide on Biosimilar Medicines for Healthcare Professionals.

[4] Halimi V, Daci A, Netkovska KA, Suturkova LJ, Babar Z, Grozdanova A (2020). Clinical and regulatory concerns of biosimilars:A review of literature. Int J Environ Res Public Health.

[5] European Commission 2019 IQVIA Report-the Impact of Biosimilar Competition in Europe.

[6] Knežević I (2011). Evaluation of similar biotherapeutics (SBPs):Scientific principles and their implementation. Biologicals.

[7] Godman B, Hill A, Simoens S, Selke G, Krulichová IS, Diaset CZ (2021). Potential approaches for the pricing of cancer medicines across Europe to enhance the sustainability of healthcare systems and the implications. Expert Rev Pharmaco Outcome Res.

[8] Wilking N, Bucsics A, Sekulovic LK, Kobelt G, Laslop A, Makaroff L (2019). Achieving equal and timely access to innovative anticancer drugs in the European Union (EU):Summary of a multidisciplinary CECOG-driven roundtable discussion with a focus on Eastern and South-Eastern EU countries. ESMO Open.

[9] Moorkens E, Godman B, Huys I, Hoxha I, Malaj A, Keuerleber S (2021). The expiry of humira®market exclusivity and the entry of adalimumab biosimilars in Europe:An overview of pricing and national policy measures. Front Pharmacol.

[10] Godman B, Wladysiuk M, McTaggart S, Kurdi A, Allocati E, Jakovljevic M (2021). Utilisation trend of long-acting insulin analogues including biosimilars across Europe:Findings and implications. BioMed Res Int.

[11] Sánchez OD, Vicens DG, Mayol AS, Pons JT (2019). Biosimilar medicines:Impact, opportunities and strategies. Twelve years of experience in Europe. Med Clin (Barc).

[12] La Noce A, Ernst M (2018). Switching from reference to biosimilar products:An overview of the European approach and real-world experience so far. EMJ.

[13] Rémuzat C, Dorey J, Cristeau O, Ionescu D, Radière G, Toumi M (2017). Key drivers for market penetration of biosimilars in Europe. J Mark Access Health Policy.

[14] European Medicines Agency Glossary-data Exclusivity.

[15] European Union Law-EUR-Lex:Regulation (EC) No 726/2004 of the European Parliament and of the Council of 31 March 2004.

[16] Kang H, Knezevic I (2018). Regulatory evaluation of biosimilars throughout their product life cycle. Bull World Health Organ.

[17] Godman B, Hill A, Simoens S, Kurdi A, Gulbinovič J, Martin A (2019). Pricing of oral generic cancer medicines in 25 European countries;findings and implications. GaBI J.

[18] Godman B, Bennie M, Baumgärtel C (2012). Essential to increase the use of generics in Europe to maintain comprehensive health care?Farmeconomia Health economics and therapeutic pathways. Farmeconomis.

[19] Al-Kinani KK, Ibrahim MJ, Al-Zubaidi RF, Younus MM, Ramadhan SH, Kadhim HJ (2020). Iraqi regulatory authority current system and experience with biosimilars. Regul Toxicol Pharmacol.

[20] Blandizzi C, Meroni PL, Lapadula G (2017). Comparing originator biologics and biosimilars:A review of the relevant issues. Clin Ther.

[21] Knežević I, Griffiths E (2011). Biosimilars-global issues, national solutions. Biologicals.

[22] Alfonso-Cristancho R, Andia T, Barbosa T, Watanabe HJ (2015). Definition and classification of generic drugs across the world. Appl Health Econ Health Policy.

[23] Shah US (2010). Chapter:Regulatory strategies and lessons in the development of biosimilars. Pharmaceutical Sciences Encyclopedia.

[24] (2019). EUR-Lex, Access to European Union low. Directive 2001/83/EC of the European Parliament and of the Council of 6 November 2001 on the Community Code Relating to Medicinal Products for Human Use.

[25] Vandenplas Y, Simoens S, Van Wilder P, Vulto AG, Huys I (2021). Informing patients about biosimilar medicines:The role of European patient associations. Pharmaceuticals.

[26] Barbier L, Simoens S, Vulto AG, Huys I (2020). European stakeholder learnings regarding biosimilars:Part I-improving biosimilar understanding and adoption. BioDrugs.

[27] Knezevic I, Griffiths E (2017). WHO standards for biotherapeutics, including biosimilars:An example of the evaluation of complex biological products. Ann N Y Acad Sci.

[28] Moorkens E, Jonker-Exler C, Huys I, Declerck P, Simoens S, Vulto Arnold G (2016). Overcoming barriers to the market access of biosimilars in the European union:The case of biosimilar monoclonal antibodies. Front Pharmacol.

[29] Ebbers CH, Schellekens H (2019). Are we ready to close the discussion on the interchangeability of biosimilars?. Drug Discov Today.

[30] Kanga HN, Knezevica I (2018). Regulatory evaluation of biosimilars throughout their product life-cycle. Bull World Health Organ.

[31] Official Gazette of Bosnia and Herzegovina Documents.

[32] Agency for Medicines and Medical Devices of Bosnia and Herzegovina Documents, Regulation.

[33] Agency for Medicines and Medical Devices of Bosnia and Herzegovina Documents, Guidelines.

[34] European Medicines Agency Similar Biological Medicinal Products.

[35] Agency for Medicines and Medical Devices of Bosnia and Herzegovina List of Medicinal Products with Marketing Authorization.

[36] WHO Collaborating Centre for Drug Statistics Methodology ATC/DDD Index.

[37] Health Insurance Fund of Republika Srpska (HIF RS) Lists of Medicine.

[38] Health Insuranceand Reinsurance Institute of the Federation of B and H (HIRI F and B) List of Medicine.

[39] Agency for Medicines and Medical Devices of Bosnia and Herzegovina Medicinal Products and Medical Devices Act (“Official Gazette of B and H, no.58/08”).

[40] Agency for Medicines and Medical Devices of Bosnia and Herzegovina. List of experts.

[41] Agency for Medicines and Medical Devices of Bosnia and Herzegovina (2011). Rulebook on Procedure and Manner of Issuing Marketing Authorization Approval (“Official Gazette of B and H, no.75/11”).

[42] U.S. Food and Drug Administration. Exclusivity and Generic Drugs:What Does It Mean?.

[43] U.S. Food and Drug Administration Guidance for Industry Reference Product Exclusivity for Biological Products Filed Under Section 351(a) of the PHS Act.

[44] Agency for Medicines and Medical Devices of Bosnia and Herzegovina (2013). Decision of Professional Board (Official Gazette of B-H number 57/13).

[45] Agency for Medicines and Medical Devices of Bosnia and Herzegovina (2018). Decision of Professional Board (Official Gazette of B-H number 75/18).

[46] Agency for Medicines and Medical Devices of Bosnia and Herzegovina Pharmacovigilance. Documents.

[47] Agency for Medicines and Medical Devices of Bosnia and Herzegovina Pharmacovigilance. Template for Adverse Drug Reaction Report.

[48] Glamoclija U, Tubic B, Kondza M, Zolak A, Grubiša N (2018). Adverse drug reaction reporting and development of pharmacovigilance systems in Bosnia and Herzegovina, Croatia, Serbia, and Montenegro:A retrospective pharmacoepidemiological study. Croat Med J.

[49] Čatić T, Begović B (2016). The attitudes of pharmacists and physicians in Bosnia and Herzegovina towards adverse drug reaction reporting. J Health Sci.

[50] European Medicines Agency Documents. Presentations.

[51] The Association of Innovative Pharmaceutical Manufacturers Official Position Regards to Biosimilar Medicinal Products.

[52] Tubic B, Marković-Peković V, Jungić S, Allocati E, Godman B (2021). Availability and accessibility of monoclonal antibodies in Bosnia and Herzegovina;findings and implications:Monoclonals in Bosnia and Herzegovina. Medicine Access@point of care. J Med Access.

[53] Leonard E, Wascovich M, Oskouei S, Gurz P, Carpenter D (2019). Factors affecting health care provider knowledge and acceptance of biosimilar medicines:A systematic review. J Managed Care Specialty Pharm.

[54] Cherny N, Sullivan R, Torode J, Saar M, Eniu A (2016). ESMO European consortium study on the availability, out-of-pocket costs and accessibility of antineoplastic medicines in Europe. Ann Oncol.

